# Late Miocene speleothems show significant warming, temperate vegetation, and wildfires in Arctic Siberia

**DOI:** 10.1038/s41598-025-12287-x

**Published:** 2025-08-04

**Authors:** Stuart Umbo, Sina Panitz, Julia Homann, Jessica McCoy, Matthew Pound, Thomas Opel, Franziska Lechleitner, Anton Vaks, Alexander Osintzev, Irina Adrian, Aleksandr Kononov, Sebastian F. M. Breitenbach

**Affiliations:** 1 Department of Geography and Environmental Sciences, Northumbria University, NE1 8ST Newcastle Upon Tyne, UK; 2 Department of Geosciences, UiT, The Arctic University of Norway, Langnes, P.O. Box 6050, 9037 Tromsø, Norway; 3 Department Chemie, Johannes Gutenberg-Universität, 55128 Mainz, Germany; 4 Alfred Wegener Institute Helmholtz Centre for Polar and Marine Research, Telegrafenberg A45, 14473 Potsdam, Germany; 5 Department of Chemistry, Biochemistry and Pharmaceutical Sciences & Oeschger Centre for Climate Change Research, University of Bern, Bern, 3012 Switzerland; 6 Geochemistry and Environmental Geology Division, Geological Survey of Israel, Jerusalem, 9692100 Israel; 7 Speleoclub Arabika, Irkutsk, 664058 Russia; 8 Lena Delta Wildlife Reserve, Tiksi, 678400 Sakha Republic Russia; 9 Irkutsk National Research Technical University, Irkutsk, 664074 Russia; 10 Institute of the Earth’s Crust, Siberian Branch, Russian Academy of Sciences, Irkutsk, 664033 Russia

**Keywords:** Climate change, Geochemistry

## Abstract

Climate driven northward boreal forest expansion into the tundra biome controlled by permafrost will play a major role in global emissions trajectories. Yet our limited understanding of the interplay between vegetation and permafrost makes predictions of changing boreal forest extent difficult. We analyse fossil pollen, stable carbon isotopes, and lignin and levoglucosan biomarkers from Tortonian speleothems (8.68 ± 0.09 Ma) from the Lena River Delta (N72.27°, E126.94°) in Arctic Siberia to infer palaeotemperature, precipitation, vegetation and fire regimes. The Tortonian provides a potential analogue for near future climate warming under extreme emissions scenarios, with global mean global temperature ca. 4.5°C above modern and atmospheric CO_2_ concentrations similar to present. We find evidence for a mixed forest regime, capable of maintaining wildfires, in a region currently dominated by tundra. Future transition to a similarly temperate regime would have large-scale impacts on the global carbon cycle.

## Introduction

Boreal forests are the largest pool of living biomass on the Earth’s surface, comprising 30% of global forested area^1^ and around 8–10 of the Earth land surface^2^. These northern forests provide extensive ecosystem services including food, timber, and leisure, and constitute a significant economic resource, contributing a third of global sawn wood lumber^3^. Globally, boreal regimes store between 367.3 and 1715.8 gigatonnes of carbon (GtC)^4^ and absorb ca. 20% of the 2.41 GtC year^− 1^ sequestered by forests^5^. Over the coming century, boreal forests are likely to experience the largest temperature increase of any forest biome^6^. The response of this region to ongoing anthropogenic warming will significantly impact local communities and the global carbon cycle.

Recently, Armstrong McKay et al. (2022)^7^ identified the expansion of northern boreal forest as a potential climatic tipping element, likely to become irreversible at ca. 4 °C of global warming above pre-industrial. CMIP5 models anticipate a slightly higher temperature threshold, with abrupt and irreversible change by the end of century for only the most extreme emissions scenarios^8^. Satellite observations show that expansion of the boreal ecotone into the tundra biome is already underway^9^. Thawing permafrost is allowing the establishment of new vegetative regimes, with woody vegetation increasing in ice-poor uplands and river valleys, and replacement of woody vegetation with (aquatic) graminoids in ice-rich lowlands^9^. Whilst this northward expansion of boreal forest into the tundra biome is anticipated to sequester ca. 6 GtC over the coming century^7^ such “Arctic greening” alters albedo in high latitudes, shifting the energy budget and driving regional temperature increases^10 ^which can impact the permafrost that underlies the tundra biome^9^. In winter, the boreal vegetation canopy traps snow, which insulates the ground from the cooling effect of the atmosphere^11^ and, where vegetation protrudes above the snowpack, it acts to reduce albedo and drive further warming^12^. Boreal expansion has the opposite effect in summer, with the vegetation canopy intercepting incoming radiation and reducing ground heat flux^13^. In general, winter warming effects tend to outweigh summer cooling^11^. Best estimates predict a net global mean surface warming of 0.14 °C per °C of global warming from tundra greening^7 ^however, uncertainty in both tipping thresholds and the resultant impact on temperature remains high. Improving our understanding of the interaction between permafrost and vegetation, alongside more realistic simulations of changing ecotone boundaries is imperative for constraining climatic projections from Earth system models^9,14^. This is particularly true in the understudied permafrost regions of coastal Siberia^9^ where most of the boreal-tundra ecotone is located.

Northward boreal expansion will play a key role in driving future wildfire prevalence through increasing fuel supply. Fire has been largely absent from the tundra biome during the last 5 ka^15^ but has increased over recent decades^15,16^. In upland tundra ecosystems, biomass burning is linked to subsequent shrub expansion^17^. The opposite is true in lowlands, where fire-driven ground heating can trigger thermokarst^18 ^waterlogging and suppression of shrub growth^17^. Wildfire is associated with permafrost thaw, driving emissions in a positive feedback cycle. Recent estimates suggest carbon emissions from biomass burning in Canada in 2023 were equivalent to fossil fuel emissions from major industrialised nations^19^. Incorporating wildfires and their impact on thermokarst into permafrost models remains a crucial missing component for constraining projections of future Arctic greenhouse gas emissions^14^.

**Fig. 1:**
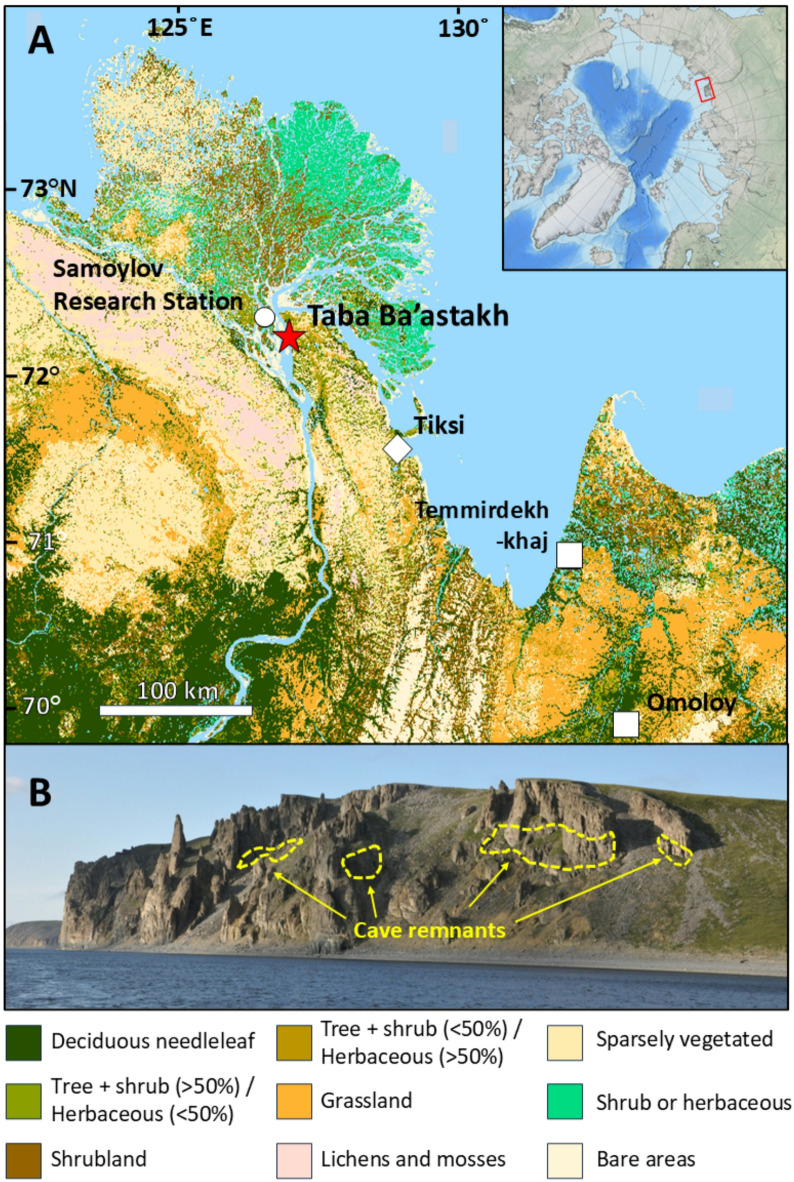
(**A**) Location of the Taba Ba’astakh sampling site (red star), Samoylov Research Station, Tiksi, and relevant Miocene palaeoclimate reconstruction sites: Temmirdekh-khaj and Omoloy from Popova et al. (2012)^49^, overlain with vegetation type^64^ (Map created in Esri ArcMap version 10.5.1, copyright Esri, Garmin, GEBCO, NOAA NGDC, and other contributors), (**B**) Photograph of the Taba Ba’astakh cliffs (approx. height is 120 m) with locations of cave remnants outlined in yellow dashed lines. (adapted from Vaks et al., 2025^56^).

Looking to warmer-than-present intervals of the Earth’s history can inform on vegetation expansion thresholds and vegetation-permafrost interactions. Here we present two speleothems (carbonate cave deposits), STBB I – 6 and STBB II – 7, from Taba Ba’stakh in the Siberian Arctic (Fig. 1) – a region largely unexplored in the literature^9^ – to infer vegetation and wildfire regimes during the late Miocene (8.68 ± 0.09 Ma, Tortonian stage). Mean global temperatures during the Tortonian were ca. 4.5 °C above modern^20 ^analogous to end-of-century warming projected under high emissions scenarios^21^. Atmospheric carbon dioxide concentrations of 400–600 ppm^22^ were comparable to those predicted for the coming decades at current emission rates^23^ and the palaeo-latitude of northern Siberia was similar to the present day^24^. We utilise speleothem-derived fossil pollen, lignin oxidation products (LOPs), levoglucosan, and stable carbon isotope ratios to infer climate, vegetation regime and palaeo-wildfire prevalence during the Tortonian.

## Results

### Vegetation reconstructions

**Fig. 2:**
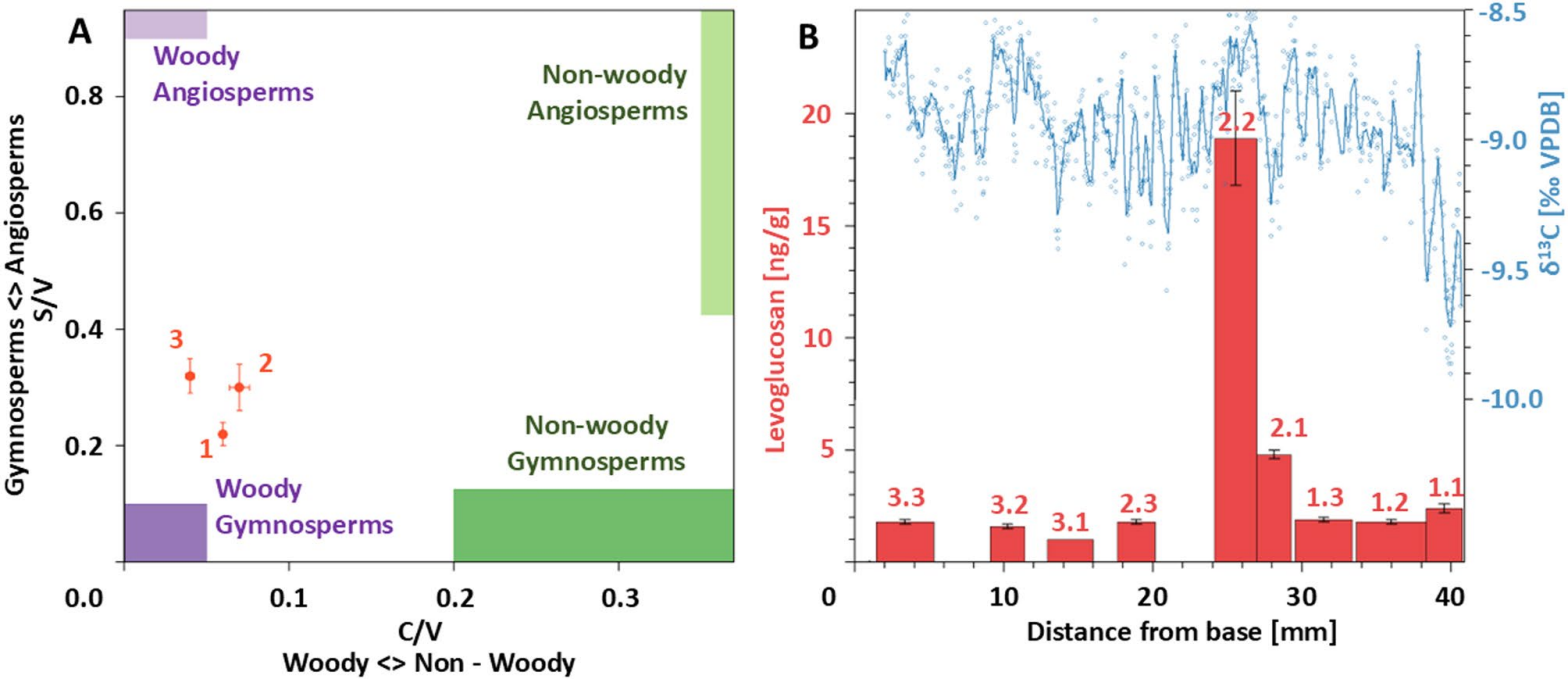
Lignin, levoglucosan and stable carbon isotope results from sample STBB II – 7. (**A**) Lignin oxidation product (LOP) ratios (S/V vs. C/V). The shaded areas indicate experimentally determined LOP ratios associated with differing plant functional types^26^. (**B**) STBB II – 7 levoglucosan concentration plotted alongside δ^13^C^13^ (**C**), smoothed with a 0.25 mm running mean. Levoglucosan error bars are one standard deviation. Levoglucosan numbering corresponds to sample numbers indicated in (A) (i.e., sample 1 in (**A**) is an amalgamation of levoglucosan samples 1.1, 1.2, and 1.3 in (B), and so on for 2 and 3). Sampling positions are shown in Fig. 3.

Stable carbon isotope ratios (δ^13^C) from sample STBB II – 7 vary between − 9.9 and − 7.4‰ VPDB, averaging − 8.9‰ VPDB (Fig. 2). Fluctuations of ca. 1‰ occur at cm scales, before a sharp decline is observed at ca. 37 mm from the base which continues until the end of the record. Low δ^13^C values, generally between − 14‰ and − 6‰, are indicative of a predominant influence of respired carbon from C3 vegetation and its soil microbial community^25^.

Pollen analysis on sample STBB I – 6 yielded a total of 29 pollen grains, of which ten were identified at family level, 16 at genus level and one with affinities to a modern species (Table 1). A further two pollen grains could not be identified due to poor preservation. Softwood gymnosperms (non-flowering plants - Pinaceae and Cupressaceae, including *Juniperus)* are most abundant with ten grains, with *Pinus* being the most prevalent genus. Hardwood angiosperms (flowering plants), particularly *Quercus*, are also highly represented with 8 grains.

Lignin analysis on sample STBB II – 7 yielded high LOP concentrations between 24 and 60 ng/g, achieving measurement uncertainty of less than ± 1.7 ng/g (Fig. 2). LOPs can be categorised into three groups, defined by their phenolic structures: vanillyl (V), syringyl (S) and cinnamyl (C). Since different plant functional groups produce lignin types in differing quantities, the ratios of these three LOP groups incorporated within the speleothem matrix provide a tool for identification of vegetation type from which they arose^26^. High C/V ratios are associated with non-woody plants since they produce significant amounts of the two cinnamyl phenols, whereas the syringyl group is found exclusively in angiosperms (flowering plants). The STBB II – 7 LOP ratios are defined by relatively low C/V (< 0.1) and S/V (< 0.4) values, typically of woody gymnosperms^27^ (non-flowering plants, e.g. conifers) (Fig. 2).

### Wildfire reconstructions.

Levoglucosan concentrations remain relatively constant (ca. 2 ng/g) along the entire STBB II – 7 growth profile, except for a large positive excursion (18.9 ng/g) between 24.1 and 27.0 mm (2.2 in Fig. 2B). Sample recovery was assessed from the concentration of a ^13^C_6_ spike which was added to each sample during preparation. Of the nine samples measured, seven recovered a ^13^C_6_-levoglucosan spike within one standard deviation of the mean for the whole dataset indicating good sample recovery. Of the two samples that yielded a reduced signal, the first, between 12.9 and 16.0 mm (3.1 in Fig. 2B) also recorded the lowest levoglucosan concentration in the entire record, suggesting that this measurement may be skewed due to poor sample recovery. However, the levoglucosan spike between 24.1 and 27.0 mm also yielded reduced ^13^C_6_ recovery and thus it is reasonable to assume that the positive levoglucosan excursion is of even greater magnitude.

Since levoglucosan is produced solely by combustion of cellulose^28^ its continual presence in the STBB II – 7 record suggests that the Tortonian vegetation regime at Taba Ba’astakh was sufficient to support wildfires^29^. The higher levoglucosan level between 24.1 and 27.0 mm is interpreted as signal of increased burning during this interval.

### Pollen based climate reconstructions

Pollen-based climate reconstructions (Table 2) give a mean annual air temperature (MAAT) of 9.6 (+ 4.5/-3.0) °C. Warm/cold season temperature reconstructions are 16.7 (+ 4.7/-2.1)°C and 2.9 (+ 4.2/-4.6)°C respectively, with a mean annual precipitation of 669 (+ 367/-212) mm (all uncertainties stated to the 50% confidence level). Modern meteorological data is available from the nearby Samoylov Research Station (N72.37°, E126.48°), 20 km to the northwest of Taba Ba’astakh (Fig. 1) which sees MAAT of -12.3 °C (1998–2017). The July average is 9.5 °C, and February average − 32.7°C^30^. Mean annual precipitation, measured at the nearby Tiksi Meteorological station, is 309 mm (1980–2018 average)^31^.

## Discussion

### Tortonian vegetation at Taba Ba’astakh

Multiple lines of evidence (δ^13^C, fossil pollen, and LOPs) indicate the presence of a mixed forest regime in Arctic Siberia during the Tortonian. The modern treeline extends to ca. 72°N, around 20 km south of our study site, and is dominated by conifer species including larches (*Larix sibirica* and *L*. *dahurica* Turcz), and pine (*Pinus sibirica* Du Tour, and *P. pumila*)^30^. No trees are found directly above the caves. Our results not only suggest that the Tortonian treeline extended at least 20 km further north compared to modern day but also reveals the presence of temperate tree species such as *Alnus*, *Cornus*, and *Quercus*.

Pollen within caves has been shown to be representative of local vegetation regimes^31,32 ^and is well preserved in speleothem calcite, with limited preservation bias^33^. Thus, we assume our record to be representative of local vegetation. This is supported by the Tortonian Temmirdekh-khaj fossil seed flora, 250 km southeast of Taba Ba’astakh (Fig. 1), that contains more than 130 taxa including many identified in the present study (*Alnus*, Caprifoliaceae, Cornaceae, Cyperaceae, and *Pinus*)^34^. Whilst *Quercus* was not identified at Temmirdekh-khaj, it has been identified at other high latitude sites in Eastern Siberia during the late Miocene^35^. The presence of temperate angiosperm species (*Alnus*, *Cornus*, and *Quercus*) suggests the Tortonian vegetation was significantly richer than modern boreal forests which tend to be dominated by gymnosperm species^36^.

Since large samples are required for pollen analysis, our record represents an integration of all taxa during the speleothem deposition period, and we are unable to distinguish whether the identified species coexisted or were present at different times. Here, we make the assumption that our assemblage provides a representation of species present throughout the entire speleothem formation period. Prior trace element analysis has shown growth rates of ca. 200 μm in STBB II − 7^39^ which correspond to growth periods of ca. 200 and 400 years for STBB II – 7 and STBB I – 6 respectively. Our data is comparable to other pre-Quaternary palaeobotanical reconstructions that generally have millennial scale sampling resolution or dating uncertainty at the 100 ka timescale^37^. We stress that the small number of pollen grains extracted from the speleothem, which falls short of the minimum 300 commonly utilised to reconstruct past vegetation assemblages^38 ^limits the statistical robustness of our pollen reconstruction. We acknowledge the limitations of the crestr approach that we employ (see methods), which infers palaeoclimatic conditions by comparison of fossil to modern taxa (modern analogues). Reconstructions over million-year timescales may be hindered by species evolution or extinction. We must also consider the taphonomic processes that transport pollen into speleothems. Dickson et al., (2023)^33^ demonstrated that the majority of fossil pollen in Australian speleothems is wind blown into caves and our fossil pollen record is predominantly derived from anemophilous (wind-blown) taxa. Sinking streams and animals can also contribute to the pollen deposited in a cave^39^ and these processes may contribute to the occasional entomophilous (insect transported) pollen grain recorded in Taba Ba’astakh (Table 1) and other speleothems from a temperate climate^40^. The dominance of anemophilous pollen is known to hinder paleoenvironmental reconstructions of species richness from fossil assemblages^41^ and we cannot rule out such biases within our fossil record. For instance, *Pinus* is a known extra-regional pollen grain in the contemporary pollen rain of the Arctic^42^. The modern treeline lies ca. 20 km south of Taba Ba’astakh (Fig. 1) with prevailing southerly winds^43^ making long distance transportation to our study site possible. Bird migrations have also been proposed as a possible pathway for pollen to enter Arctic lakes^42^ and could account for the presence of taxa more typical of warmer climates, but we deem it relatively unlikely that such a signal would significantly influence our speleothem pollen record.

Vegetation reconstructions from high latitudes are rare during the Tortonian^44,45^. Using a best-fit climate vegetation model, Pound et al. (2012)^45^ suggested that evergreen boreal forest may have extended as far north as 80°N and was more species diverse than present-day boreal forest, encapsulating our site. Our results lend support for this northerly ecotone shift but suggest additional presence of temperate species (including *Alnus*,* Cornus*,* Daphne*,* Oleaceae* and *Quercus*) that are sparce in modern boreal forest regimes which are dominated by *Pinus*,* Abies*,* Larix*, and *Picea*^46^. Plant and seed remains from Temmirdekh-khaj (132°E, 71°N) and Omoloy (133°E, 70°N), ca. 100 km and 250 km southeast of Taba Ba’astakh, respectively^47^ (Fig. 1), as well as model-derived biome reconstructions^45^ support our findings, with evidence of mixed forest with broadleaf and needleleaf evergreen species. To our knowledge, Taba Ba’astakh provides the highest latitude continental vegetation reconstruction during the late Miocene. Our LOP and pollen reconstruction suggests a mixed forest dominated by conifers and enriched by more temperate angiosperm tree species at the northern extreme of the Eurasian landmass.

### Tortonian climate reconstructions

Our MAAT reconstructions are in good agreement with previous work from Umbo et al.^48^ which derived a Tortonian MAAT between 6.1 and 11.1 °C at Taba Ba’astakh using clumped isotope and fluid inclusion analysis of speleothems. Additionally, our pollen-derived MAAT estimate agrees with late Tortonian MAAT derived from fossil plant remains from nearby Temmirdekh-khaj and Omoloy (Fig. 1)^49^ (9.3–10.8 °C and 7.3–16.1 °C respectively). Whilst more taxa would potentially have improved the uncertainty of our reconstruction, any change outside of the model estimates would be purely speculative. It is also worth noting that despite the low numbers of grains, both anemophilous (wind pollinated) and entomophilous (insect pollinated) plant taxa are represented in the assemblage, which would suggest that potential pollination syndrome biasing in the pollen data is not controlling the assemblage.

Our seasonal reconstructions compare well with those from Temmirdekh-khaj and Omoloy^49 ^with a slight offset since our record reconstructs warmest and coldest quarters while the Temmirdekh-khaj and Omoloy records reconstruct the warmest and coldest month. In addition, it is also known that the co-existence approach employed to produce the Temmirdekh-khaj and Omoloy palaeotemperature estimates^49^ produces higher temperature estimates than the crestr model, employed in this study, due to a more limited modern dataset and lack of statistical diagnosis^50^. Our warm season temperature estimate of 16.7 (+ 4.7/-2.1 °C) is slightly cooler than the warmest month reconstructions from Temmirdekh-khaj (21.6–23.8 °C) and Omoloy (21.7–25.6 °C)^49^. Similarly, our cold season reconstruction of + 2.9 (+ 4.2/-4.6 °C) aligns with the coldest month estimates of -2.8–1.1 °C and − 3.8–6.4 °C, respectively^49^. Taken together these results suggest a reduced temperature seasonality at Taba Ba’astakh compared with the more southerly sites^49^ which we attribute to the proximity of Taba Ba’astakh to the Laptev Sea.

Our late Miocene mean annual precipitation estimate of 669 (+ 367/-212) mm is considerably higher than the 309 mm observed today^51^ and confirms a considerably wetter Tortonian in Arctic Siberia found in the Temmirdekh-khaj and Omoloy records (735–975 mm and 592–1206 mm per year respectively)^49^. Higher precipitation was likely driven by increased evaporation and moisture transport from the Arctic Ocean which was largely ice-free during Tortonian summers^52^.

### Implications for future anthropogenic climate change

Our pollen-based temperature reconstruction of 9.6 (+ 4.5/-3.0)°C, ca. 20 °C above present day, provides an estimate of warming at our study site given future global mean surface temperature rises similar to those of the Tortonian (ca. +4.5 °C above modern)^20^. Our Arctic warming estimate (20 °C above present) is considerably higher than model projections of ca. 10–12 °C of warming by 2100 under high emissions scenarios^53,54 ^ and whilst our temperature reconstructions can inform and constrain such projections, we emphasise that they should be viewed in the context of different climatic boundary conditions during the Tortonian, compared to modern day. For example, the Greenland Ice Sheet was largely restricted to the northern and eastern landmass^55^, Eurasia was likely free from permafrost^56^, and the Arctic Ocean was ice-free during summers^52^. Such planetary scale differences in environmental conditions would have considerably altered the Miocene global energy budget but are unlikely to drive decadal to centennial scale temperature changes in the near future.

Our Tortonian vegetation reconstruction provides context for possible future vegetation shifts at high latitudes. We present paleoclimatic evidence of temperate forest at the northern extremes of the Eurasian landmass. This suggests that runaway tundra greening, estimated to occur at global temperatures of ca. +4 °C above pre-industrial^7^, is likely to have occurred given ca. +4.5 °C of global terrestrial warming during the Tortonian. The presence of mixed forest in a region dominated by tundra in the modern day, suggests a continuation of the ongoing northward migration of boreal forests observed over recent decades^57,58^. Landsat imagery from 1999 to 2014 shows that the Lena Delta is already undergoing regional greening^59 ^and our findings suggest this trend may continue with increasing atmospheric temperature. Gymnosperm dominated boreal biomes at high latitudes will likely experience increasing presence of more temperate angiosperm species including *Alnus* and *Quercus* as global, and particularly Arctic, temperatures rise.

Vaks et al. (2025)^56^ recently suggested that speleothem formation at Taba Bastaakh during the Tortonian was indicative of surface permafrost absence which allowed liquid water to penetrate the cave environment and facilitate carbonate deposition. Modern observations of Siberian coastal lowlands suggest expansion of shrub vegetation typically occurs following abrupt permafrost thaw^9^ and thus the presence of woody vegetation at our site, alongside our reconstructed MAAT of 9.6 (+ 4.5/-3.0) °C, lends support to these findings. Given Taba Ba’astakh’s location at the northern limit of modern Eurasian permafrost, this implies permafrost degradation across most of the northern Hemisphere. Boreal permafrost thaw is a potential climate tipping element which, once surpassed could produce between 43 –and 350 GtC of emissions^7^. Estimates of the permafrost tipping thresholds are between 4 °C and 6 °C^7,60 ^although recent modelling has suggested permafrost degradation may exhibit a more linear response to atmospheric warming^61^.

If near future boreal regimes expand into modern northern permafrost regions, as we show here for the Tortonian, it is likely that a proportion of greenhouse gas emissions from permafrost thaw may be offset by vegetative carbon uptake. Biogeochemical modelling suggests that vegetation expansion under focused mitigation pathways will largely offset emissions from permafrost thaw, changing the Arctic into a net carbon sink^62^. However, under more extreme emission scenarios, with temperature rises similar to those of the Tortonian, carbon emissions outpace vegetation uptake, and the Arctic becomes a net source of carbon^62^. Quantifying the magnitude of net emissions remains difficult due to the large spread in model projections for CO_2_ uptake, arising in large part from an uncertain vegetation response to warming^14^. Our findings suggest a proliferation of temperate species into high latitude permafrost regions under global mean temperature increases similar to those observed in the Tortonian.

Whilst we are unable to deduce absolute fire prevalence, the presence of levoglucosan across the entire STBB II – 7 record suggests regular biomass burning events in a region where they (so far) remain rare^63^. The presence of mixed woodland at our study site during the Tortonian will likely have increased fuel abundance which, alongside temperatures ca. 20 °C above present, would have provided favourable conditions for wildfires. Future wildfire prevalence and its impact on thermokarst dynamics remain a key missing element in projections of Arctic carbon emissions^14^. Observations show that wildfire prevalence in tundra ecosystems has increased over recent decades^15,16^. Our reconstructions suggest that further global mean temperature increases, up to the ca. 4.5 °C observed in the Tortonian, will drive replacement of the tundra biome with temperature forest capable of supporting sustained fire regimes.

Our Taba Ba’astakh records provide multiple lines of evidence for a temperate vegetative regime in Arctic Siberia during the Tortonian. The presence of such temperate species in a region occupied by the tundra today implies regional mean annual temperature increases of ca. + 20 °C, in line with previous temperature estimates from clumped isotope reconstructions^48 ^and absence of near surface permafrost. Thus, we conclude that extreme future emissions scenarios giving rise to Tortonian levels of surface warming would likely have significant consequences for high latitude ecosystems and the global carbon cycle.

### Display items

**Fig. 3:**
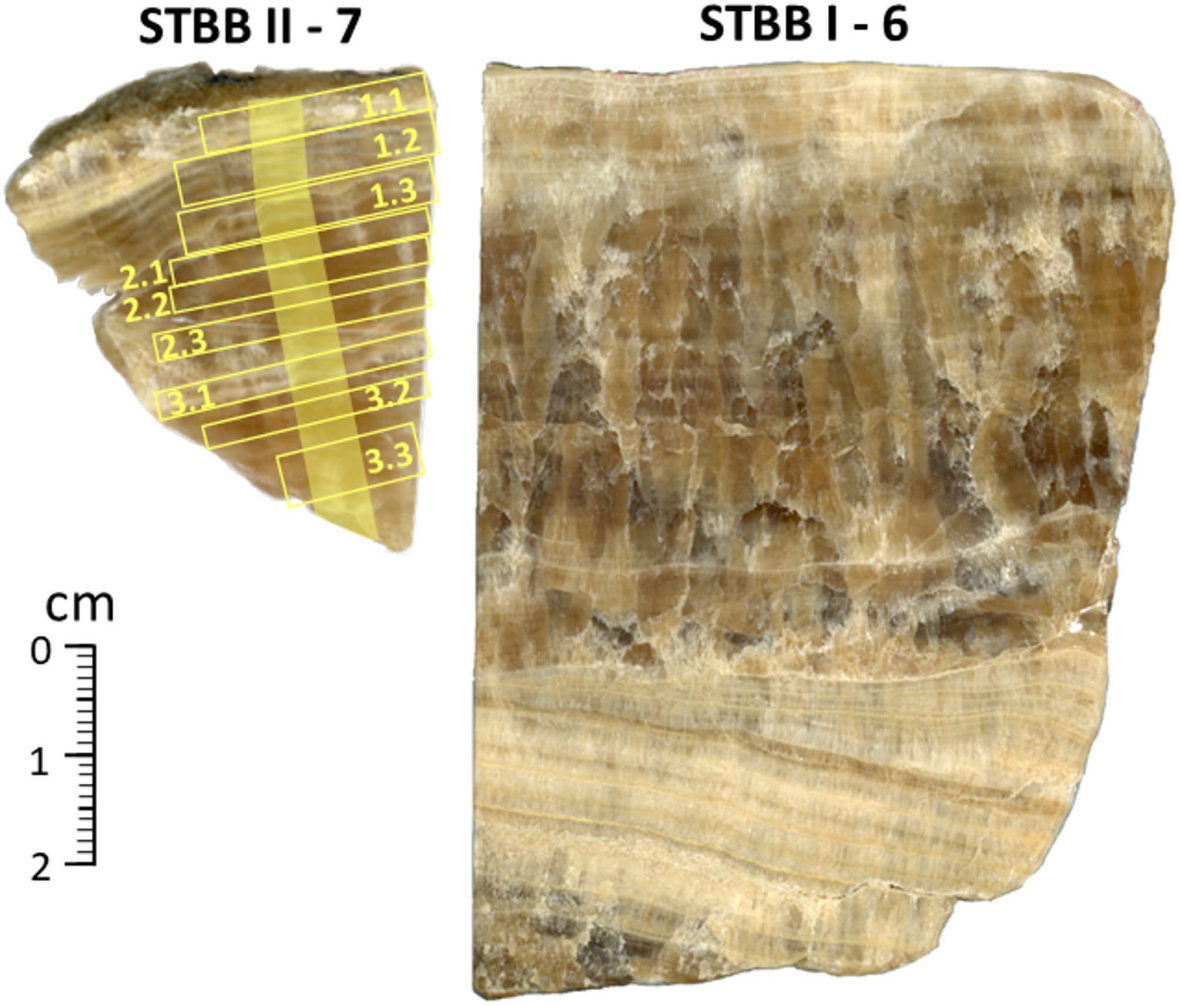
Speleothem samples utilised in this study. The yellow boxes on STBB II – 7 denote levoglucosan and lignin oxidation product sampling locations with numbering denoting sample grouping for LOP analysis (Sect. 3.2). The yellow shaded track shows the δ^13^C sampling profile.

**Table 1:** Pollen counts from STBB I – 6. All pollen grains are counted only once such that, if they are identified to the species level, they have not been additionally included at the family and genus level.

	Group	Family	Taxa	Count	Total
Woody	Hardwood angiosperms	Adoxaceae	-	1	8
		Betulaceae	*Alnus*	1	
		Cornaceae	*Cornus*	2	
		Fagaceae	*Quercus*	3	
		Oleaceae	*-*	1	
	Softwood gymnosperms	Cupressaceae	*Juniperus*	3	10
		Cupressaceae	-	1	
		Pinaceae	*Pinus*	4	
		Pinaceae	-	2	
Non-woody		Boraginaceae	-	1	8
		Thymelaeaceae	*Daphne*	1	
	Shrubs, herbs and sedges Mosses	Caprifoliaceae	*Saxifraga* aff. *hirculus*	1	
		Amaranthaceae	-	2	
		Caprifoliaceae	-	1	
		Cyperaceae	-	1	
		Primulaceae	*Androsace*	1	
		Sphagnaceae	*Sphagnum*	1	1
	Unknown	-	-	2	2

**Table 2:** Palynological climate reconstructions from the *crestr* model.

	Optima (°C)	50% conf low	50% conf high	95% conf low	95% conf high
Mean Annual Temperature	9.6	6.5	14.0	-1.6	22.2
Mean Temperature of the Warmest Quarter	16.7	14.6	21.5	9.3	28.4
Mean Temperature of the Coldest Quarter	2.9	-1.7	7.1	-14.1	16.8
Mean Annual Precipitation	669	457	1036	196	2252

## Methods

### Site and sample description

Taba Ba’astakh (N72.27°, E126.94°), is located on the banks of the Lena River Delta, ca. 100 km northwest of the settlement of Tiksi in northeastern Siberia, Sakha Republic (Fig. 1). Carboniferous-aged carbonate cliffs (Mikhaltsov et al., 2018), ca. 140 m in height, house multiple caves between 70 and 120 m above the riverbank. While ice infilling prevents access to the interior of the caves, numerous heavily eroded relic caves are found with exposed speleothems in situ along the cliff walls.

Temperature data is available from the nearby Samoylov Research Station (N72.37°, E126.48°), 20 km to the northwest of Taba Ba’astakh which sees mean annual air temperature of -12.3 °C (1998–2017). The July average is 9.5 °C, and February average − 32.7 °C (Boike et al., 2019). Mean annual precipitation, measured at the nearby Tiksi Meteorological station, is 309 mm (1980–2018)^51^. Taba Ba’astakh is characterised by a polar tundra climate (ET according to the Köppen-Geiger classification)^65^.

Fourteen speleothem samples were collected from Taba Ba’astakh, from which 66 subsamples were dated with U-Pb techniques, according to a modified method from Vaks et al. (2020)^66^ and Mason et al. (2022)^67^. These dates yielded a mean age of 8.68 ± 0.09 Ma^56^. The ^235^U/^207^Pb and ^208^Pb/^207^Pb ratios and their uncertainties of speleothems STBB I – 6 and STBB II – 7 which were selected for this study are located on this isochron line and therefore we adopt the 8.68 ± 0.09 Ma age. Construction of age-depth models along sample growth profiles was not possible due to overlapping uncertainties in top and bottom ages. Prior X-ray Diffraction (XRD) analyses had shown all samples to be calcite^56^.

### Pollen analysis

Sample STBB I – 6 was selected for pollen analysis due to its dark colour suggesting a higher organic content, and its large size providing a sufficiently large quantity of material. Pollen concentration is generally low in speleothems and large samples are required to provide sufficient counting statistics^68^. Samples were analysed at Northumbria University using an adapted methodology from Sniderman et al. (2016)^69^. Sixty grams of STBB I – 6 were rinsed in deionised water prior to surface etching in 1.5% HCl for ten minutes to remove post-depositional contaminant pollen. Samples were dissolved completely in 37% hdrochloric acid and two lycopodium tablets added to provide confirmation of final pollen yield from the dissolved sample. The resultant solution was sieved to obtain the 10–125 μm fraction of residual material. Non-pollen particulate matter was then removed using a sodium polytungstate heavy liquid solution made to specific gravity 2.0 to separate pollen and particulate based on buoyancy. Pollen grains were removed from the supernatant surface with a pipette and detrital matter, which had sunk to the base of the container, was discarded. The sample was then submerged in 10% KOH solution at 80 °C for 3 min to reduce clumping of organic matter. After each stage, samples were rinsed in deionised water to remove chemical residue and centrifuged at 3300 rpm for three minutes before decanting supernatant waste. Finally, samples were dehydrated in isopropyl alcohol and suspended in silica oil, before mounting onto microscopy slides. Analysis of marble samples prepared using the same methodology revealed zero contaminant pollen specimens.

Fossil pollen types were identified and assigned their nearest living relative (NLR) by comparison to published literature and reference materials. We incorporated family and genus level NLRs into the Bayesian probability density function-based reconstruction model, *crestr* R package (available at https://github.com/mchevalier2/crestr*)*, on a presence-absence basis, restricting modern climate-taxa space to Eurasia and keeping all other parameters as recommended^70^. The crestr model assumes the climatic niche of identified fossil pollen is the same as that of the NLR and uses modern plant distribution data^71^ from the Global Biodiversity Information Facility overlaid with the WorldClim 1.4 climate dataset^72^ to produce probability density function curves of NLRs. From these, a mean value with 50% and 95% uncertainty ranges was generated for the fossil assemblage^70^.

### Stable carbon isotopes

STBB II – 7 was sampled at 50 μm resolution with a Sherline micromill according to the methodology of Lechleitner et al. (2020)^73^ (Fig. 3). Stable isotope analysis was conducted using an adapted method from Spötl & Vennemann (2003)^74^ at Northumbria University. Briefly, 110 ± 10 µg of sample was reacted with concentrated orthophosphoric acid in a 12 ml borosilicate exetainer tube heated to 70 °C in a helium environment. Analyte gas was measured on a ThermoScientific Delta V Isotope Ratio Mass Spectrometer coupled with a ConFlo IV. An in-house laboratory carbonate standard (Plessen) and international standards NBS18 and IAEA603 were measured every 10 samples to correct for instrumental drift, matrix effects, and project onto the VPDB reference frame. We used an in-house standard (Pol-2) to evaluate long-term reproducibility, achieving an external standard deviation of < 0.08‰ for δ^13^C.

### Levoglucosan and lignin oxidation products (LOPs)

High-resolution stable isotope analysis of STBB II – 7 allowed identification of major isotopic transitions which were targeted for levoglucosan and lignin analysis. Samples of mass 400 ± 50 mg were milled along the growth length at isotopic transition intervals. Levoglucosan and lignin analysis was conducted at the Johannes Gutenberg University of Mainz according to the methodology of Homann et al. (2022)^29^. Briefly, samples were pulverised and spiked with 100 µL of ^13^C_6_ levoglucosan solution (100 ng mL^− 1^ in acetonitril) prior to a 45-minute extraction in methanol, filtering and evaporation to dryness in salinated vials. After redissolution, samples were filtered to 0.2 μm prior to measurement.

Due to the larger samples required, it was necessary to combine adjacent levoglucosan samples in triplet for LOP analysis. Polymeric lignin was extracted from the dissolved speleothem using solid phase extraction and subsequently broken down to monomeric units (LOPs) through oxidation. Oxidation was achieved by the addition of CuSO_4_ solution and heating to 155 °C for 90 min using a method adapted from Yan and Kaiser (2018)^75^. LOPs were concentrated using solid phase extraction and solutions filtered to 0.2 μm prior to analysis.

Levoglucosan and LOP analysis was conducted on a Dionex UltiMate 3000 ultra-high-performance liquid chromatography system coupled to a heated electrospray ionisation source and Q Exactive Orbitrap high resolution mass spectrometer.

## Data Availability

All datasets are available upon request from the corresponding author (stuart.umbo@northumbria.ac.uk) and will be made available on the Zenodo database pending publication of the manuscript.
